# Research Trends and Hotspots of Medical Electrical Impedance Tomography Algorithms: A Bibliometric Analysis From 1987 to 2021

**DOI:** 10.7759/cureus.49700

**Published:** 2023-11-30

**Authors:** Zhangjun Tan, Shiyue Lu, Lin Yang, Yuqing Xu, Shaojie Qin, Meng Dai, Zhe Li, Zhanqi Zhao

**Affiliations:** 1 Department of Critical Care Medicine, Renji Hospital, School of Medicine, Shanghai Jiao Tong University, Shanghai, CHN; 2 Department of Aerospace Medicine, Fourth Military Medical University, Xi'an, CHN; 3 Department of Biomedical Engineering, Fourth Military Medical University, Xi'an, CHN; 4 School of Biomedical Engineering, Guangzhou Medical University, Guangzhou, CHN; 5 Department of Critical Care Medicine, Peking Union Medical College Hospital, Beijing, CHN; 6 Department of Technical Medicine, Furtwangen University, Villingen-Schwenningen, DEU

**Keywords:** hotspot, woscc, algorithm, eit, bibliometric analysis

## Abstract

Electrical impedance tomography (EIT) is a gradually maturing medical imaging technique that relies on computational algorithms for reconstructing and visualizing internal conductivity distributions within the human body. To provide a comprehensive and objective understanding of the current state and trends in the EIT algorithm research, we conducted bibliometric analysis on a 25-year EIT algorithm research dataset sourced from Web of Science Core Collections. We visualized publication characteristics, collaboration patterns, keywords, and co-cited references. The results indicate a steady increase in annual publications over recent decades. The United States, United Kingdom, China, and South Korea contributed 60% of the articles collaboratively. Keyword analysis unveiled three distinct stages in the evolution of EIT algorithm research: the establishment of fundamental algorithm frameworks, optimization for improved imaging performance, and the development of algorithms for clinical applications. Additionally, there has been a shift in research focus from traditional theories to the incorporation of new methods, such as artificial intelligence. Co-cited references suggest that integrating EIT with other established imaging techniques may emerge as a new trend in EIT algorithm research. In summary, EIT algorithms have been a consistent research focus, with current efforts centered on optimizing algorithms to enhance imaging performance. The emerging research trend involves utilizing more diverse and intersecting algorithms.

## Introduction and background

Electrical impedance tomography (EIT) is a bioimaging technique that relies on measuring electrical impedance through body-surface electrodes. Medical EIT has been used in various clinical and experimental settings, including pulmonary, brain, and tissue monitoring [1-3].

The EIT algorithms play a crucial role in generating EIT images used in bedside applications, making it a key focus of research within the EIT field [4]. The EIT algorithms encompass tasks such as reconstructing EIT images, analyzing electrical properties of tissues, calculating sensitivity matrices, and interpreting images [5]. Notably, recent advancements in reconstruction algorithms have received significant attention [5,6]. An objective review of the current research status on EIT algorithms would aid researchers in acknowledging the progress achieved in this field, identifying the scientific focus, promoting further interdisciplinary collaboration, and expanding clinical applications [7].

The bibliometric analysis offers a feasible approach for qualitatively and quantitatively evaluating publications on a specific topic through machine learning. The evaluation includes productivity, international cooperation, identification of hot spots, and emerging trends. Several studies have utilized bibliometric analysis to construct a research map of EIT clinical applications and hardware [3,8-10]. However, a specific bibliometric analysis of the topic of the EIT algorithm has not yet been conducted. The present study aims to investigate and visualize the knowledge map of the current status, emerging research areas, and future trends in medical EIT algorithms through bibliometric analysis to provide a comprehensive understanding.

## Review

Materials and methods

Objects and Retrieval Strategies

The Web of Science Core Collection (WoSCC) database is one of the most comprehensive, systematic, and authoritative databases, encompassing over 12,000 influential journals from around the world [11]. For our research, we extracted the complete dataset from the WoSCC. The search strategy employed was as follows: TS = ("Electrical Impedance Tomography") AND (("algorithm") OR ("reconstruction")), with a time window from January 1, 1987, to December 31, 2021. We limited the publication type to include "Article," "Review," "Letter," and "Proceeding paper" and restricted the language to English. All investigators gathered the literature on January 23, 2021, to mitigate any potential bias due to database updates.

Data Collection

All the results were independently searched by two investigators, and there was a 98% agreement between their findings, indicating a significant level of consistency. Publications that were deemed less relevant to the algorithm or reconstruction topic were excluded based on the judgment of at least two experienced experts, such as publications in the fields of materials science and technology (such as materials science ceramics and materials science textiles), chemistry (including thermodynamics, engineering, and petroleum), physics (including mechanics and engineering aerospace), geography (including engineering geological, mining mineral processing, construction building technology, meteorology atmospheric sciences, and water resources), agriculture, forestry, animal husbandry, and environmental sciences. The data was exported in both text format and Unicode Transformation Format-8 (UTF-8) to facilitate further software analysis.

Bibliometric Analysis by WoSCC Output

The primary functions of the WoS core database and Microsoft Excel (Microsoft 365; Microsoft, Redmond, Washington) are to generate and present various characteristics of publications. These characteristics include the number of literature items, publication years, countries, institutions, journals, citations, etc.

Bibliometric Analysis by VOSviewer

We utilized VOSviewer, a bibliometric software version 1.6.16 (Leiden University, Leiden, the Netherlands, https://www.vosviewer.com/), to process the data and generate a co-authorship network map depicting the relationships between countries, authors, and institutions as well as the co-occurrence map of keywords [12]. Certain thresholds were set, including a minimum publication count of five for collaborative countries, authors, and institutions and a minimum co-occurrence count of five for the co-occurrence network of keywords. To enhance the efficiency of our analysis, we merged different keywords that had similar meanings. For example, "eit," "electrical impedance tomography (EIT)," "electrical-impedance tomography," and "impedance tomography" were consolidated into the single term "electrical impedance tomography."

Bibliometric Analysis by CiteSpace

CiteSpace, a bibliometric software, version 5.7R5 (Chen Meichao, Drexel University, https://citespace.podia.com/), was utilized for conducting keyword burst analysis as well as cited reference burst, cluster, and timeline view analysis.

Results

Publication Output of WoSCC

**Figure 1:**
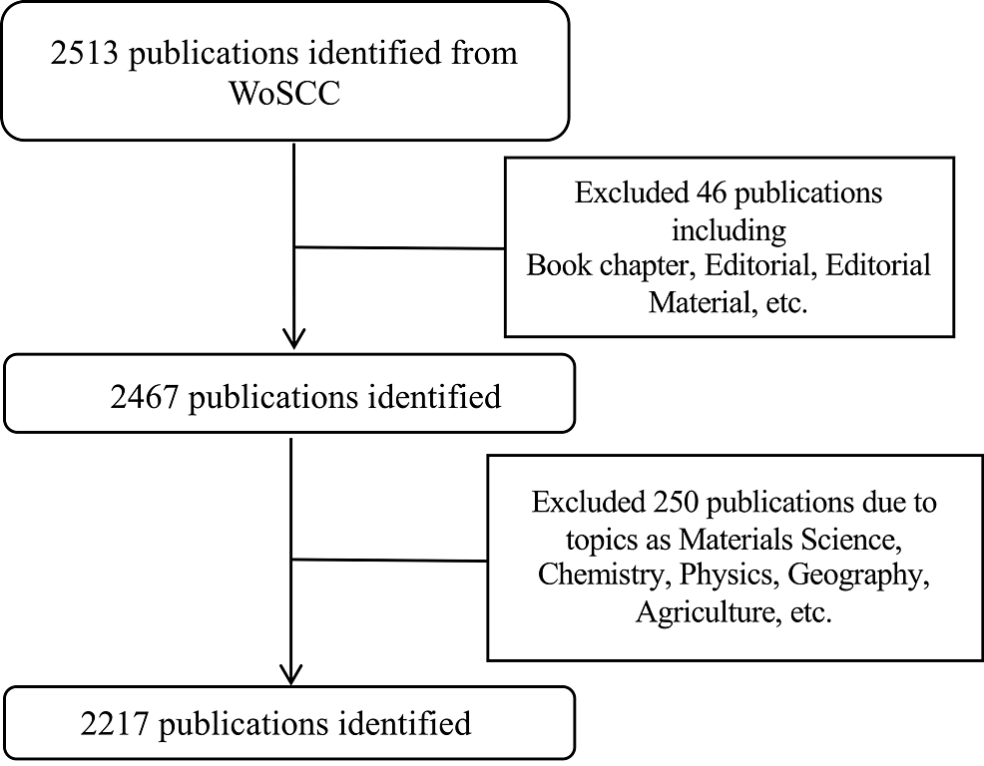
Flow chart of included publications WoSCC: Web of Science Center.

According to the search strategy, a total of 2513 publications were identified. After filtering, we analyzed 2217 research articles, reviews, letters, and proceeding papers published in English and focused on the EIT algorithm (Figure 1).

Growth Trend and Geographical Distribution

**Figure 2:**
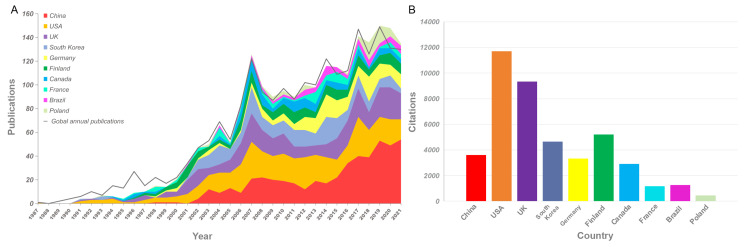
Global and the top 10 countries’ annual publications (A) and citations (B) on EIT algorithms between January 1, 1987, and December 31, 2021 The gray line (A) indicates the global publication numbers. The color indicates countries, and the plate size indicates publication numbers (A) and citations (B) of the top 10 highly productive countries. The total number of publications from the top 10 countries may exceed the overall total due to the inclusion of publications with intercountry cooperation, which were counted more than once in the statistics. Image credits: Authors of this study.

Publications on the EIT algorithm from 1987 to 2021 were available in 63 countries. The annual global publications on the EIT algorithm showed a generally increasing trend and exceeded 100 for the first time in 2007 (Figure 2). The top three most prolific and cited countries were China, the United States of America (USA), and the United Kingdom (UK).

Most Productive Institutions, Authors, Journals, and Highly Cited References

The top 10 institutions, authors, and journals that published the most pieces of literature on EIT algorithms are summarized in Tables 1-3, respectively. In our study, universities (described as univ; publication numbers; and citation numbers) are the main types of institutions. Kyung Hee University (Kyung Hee Univ; 122; 2960), Tianjin University (Tianjin Univ; 121; 938), and Yonsei University (Yonsei Univ; 89; 2844) ranked top three in the number of publications. The *Journal of Physiological Measurement* (*J Physiol Meas*; 269; 6,522) published the highest number of EIT publications and owned the most frequently published articles. In addition, a total of 338 authors have contributed to related articles, with Woo EJ, Seo JK, and Kwon O from South Korea emerging as the top three most prolific authors. The top 10 cited references included two reviews and eight articles (Table 4), and the article by Cheney et al. [13] was the most cited.

**Table 1:** Top 10 prolific institutions of EIT algorithm research UCL: University College London; EIT: Electrical impedance tomography.

No.	Institute	Publication	Citation
1	Kyung Hee Univ	122	2960
2	Tianjin Univ	121	938
3	Yonsei Univ	89	2844
4	UCL	88	3262
5	Univ Manchester	75	3577
6	Konkuk Univ	71	2073
7	Carleton Univ	55	1429
8	Fourth Mil Med Univ	51	346
9	Middlesex Univ	48	1611
10	Dartmouth Coll	47	1389

**Table 2:** Top 10 prolific authors of EIT algorithm research *Citation: The cumulative citation count for all publications by the author. EIT: Electrical impedance tomography.

No.	Author	Publication	Citation*
1	Woo and Seo	120	3206
2	Seo et al.	78	2672
3	Kwon et al.	70	2227
4	Adler et al.	67	2376
5	Wang et al.	50	485
6	Dong F	45	308
7	Bayford RH	44	1258
8	Vauhkonen et al.	43	2282
9	Rymarczyk T	43	256
10	Fu F	38	238

**Table 3:** Top 10 prolific journals of EIT algorithm research *Physiol Meas: Physiological Measurement; Inverse Probl: Inverse Problems; IEEE T Med Imaging: IEEE Transactions on Medical Imaging; Meas Sci Technol: Measurement Science and Technology; IEEE T Bio-Med Eng: IEEE Transactions on Biomedical Engineering; Phys Med Biol: Physics in Medicine and Biology; IEEE Sens J: IEEE Sensors Journal; IEEE T Instrum Meas: IEEE Transactions on Instrumentation and Measurement; Inverse Probl Imag: Inverse Problems and Imaging; Inverse Probl Sci En: Inverse Problems in Science and Engineering. #IF: Impact factor. EIT: Electrical impedance tomography.

No.	Journal*	Publication	Citation	IF^#^
1	Physiol Meas	269	6522	2.83
2	Inverse Probl	128	4219	2.41
3	IEEE T Med Imaging	103	3799	10.05
4	Meas Sci Technol	90	2378	2.05
5	IEEE T Bio-Med Eng	75	3416	4.54
6	Phys Med Biol	59	2586	3.61
7	IEEE Sens J	51	781	3.30
8	IEEE T Instrum Meas	46	568	4.02
9	Inverse Probl Imag	37	574	1.64
10	Inverse Probl Sci En	37	358	1.95

**Table 4:** The top 10 most cited reference* articles on EIT algorithm publications *Cited reference: The reference articles of publications on the EIT algorithm involved in the analysis. EIT: Electrical impedance tomography.

Reference title	Publication type	Author	Year	Journal	Citations	Reference
Electrical impedance tomography	Article	Cheney et al.	1999	Siam Rev	722	[13]
Electrical impedance tomography	Review	Borcea	2002	Inverse Probl	489	[14]
Uses and abuses of EIDORS: an extensible software base for EIT	Article	Adler et al.	2006	Physiol Meas	411	[15]
Comparing reconstruction algorithms for electrical impedance tomography	Article	Yorkey et al.	1987	IEEE T Bio-Med Eng	402	[16]
GREIT: a unified approach to 2D linear EIT reconstruction of lung images	Article	Adler et al.	2009	Physiol Meas	356	[17]
Imbalances in regional lung ventilation a validation study on electrical impedance tomography	Article	Victorino et al.	2004	Am J Resp Crit Care	344	[18]
Tikhonov regularization and prior information in electrical impedance tomography	Article	Vauhkonen et al.	1998	IEEE T Med Imaging	336	[19]
Calderon’s inverse conductivity problem in the plane	Article	Astala and Païvärinta	2006	Ann Math	334	[20]
Three-dimensional electrical impedance tomography	Article	Metherall et al.	1996	Nature	309	[21]
EIT reconstruction algorithms: pitfalls, challenges, and recent developments	Review	Lionheart	2004	Physiol Meas	308	[5]

Collaboration Between Countries, Authors, and Institutions

**Figure 3:**
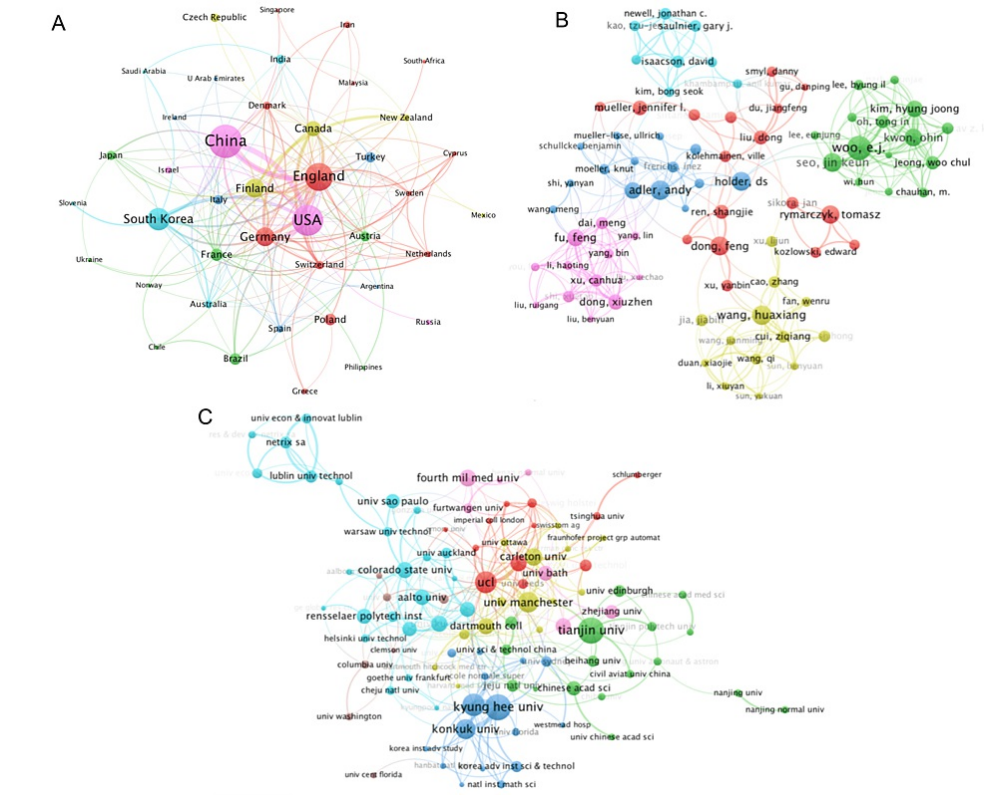
Collaborations between countries (A), authors (B), and institutions (C) on EIT algorithm research between January 1, 1987, and December 31, 2021 Different colors indicated different clusters of countries (A), authors (B), and institutions (C); color plate size indicated the publications number, and the boldness of lines between circles indicated the strength of linkage calculated on the frequency of collaborations. Image credits: Authors of this study.

The collaboration map illustrating the relationships between countries, authors, and institutions is presented in Figure 3. The visualization showcases the combined efforts, measured as total link strength, among the most prolific countries, institutions, and authors. According to our study, the USA (225), the UK (216), and China (165) emerged as the most cooperative countries in terms of the EIT algorithm. Additionally, Woo EJ (207) and Kim HJ (143) from South Korea as well as Fu F (154) and Dong XZ (132) from China were identified as the most cooperative authors worldwide, showcasing strong internal collaboration. Notably, Kyung Hee University (164), Yonsei University (136), and Konkuk University (107) were the institutions with the highest level of cooperation.

Keywords Co-occurrence Network and Overlay Analysis

**Figure 4:**
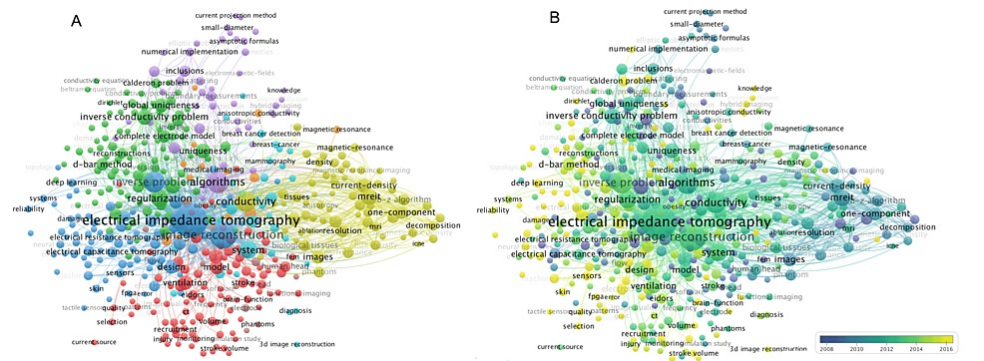
Keywords co-occurrence network map (A) and overlay analysis (B) on EIT algorithm research between January 1, 1987, and December 31, 2021 Circle size represents the number of occurrences, and the line boldness indicates link strengths. Different colors represent different clusters (A) and different average years of publications in which the keyword occurs (B), and the color of publications before 2008 in Panel B is the same as 2008 due to the limited number. Image credits: Authors of this study.

Co-occurrence network mapping and overlay analysis were conducted on 447 keywords that appeared five or more times in publications on the EIT algorithm. The keywords were categorized into eight clusters, with the five largest clusters focusing successively on "algorithm," "lung-based clinical application direction," "new algorithm reconstruction methods," "key problems in reconstruction algorithm," and "algorithm optimization." The remaining clusters were related to "extrapulmonary clinical application" and "comparison of algorithms". The main keywords in each cluster are presented in Appendix Table 7. In addition to the keywords "electrical impedance tomography," "reconstruction," and "algorithm," five other frequently co-occurring keywords were identified: "inverse problem" (316; 755), "conductivity" (273; 1882), "tomography" (195; 923), "system" (174; 1063), and "regularization" (165; 927) (Figure 4, Panel A). The top 10 most frequent keywords are listed in Table 5.

**Table 5:** Top 10 highly frequent keywords on EIT algorithm publication EIT: Electrical impedance tomography; MREIT: Magnetic resonance electrical impedance tomography.

Rank	Keywords	Occurrences	Link strength
1	Electrical impedance tomography	1524	6973
2	Image reconstruction	801	4157
3	Algorithms	429	2586
4	Inverse problem	316	1755
5	Conductivity	273	1882
6	Tomography	195	923
7	System	174	1063
8	Regularization	165	927
9	MREIT	135	968
10	Electrode models	180	619

To further explore the trends in these keywords, an overlay network was created (Figure 4, Panel B). The earliest and latest keywords, along with their average publication year, are summarized in Table 6. The keyword "applied potential tomography" (2003.08) was the earliest, while "deep learning" (2020.06) was the latest. Among the earliest frequent keywords, "boundary-value problem" (2007.64) had the highest co-occurrence frequency, while "machine learning" (2018.81) ranked first among the latest frequent keywords.

Keywords Burst Analysis

**Figure 5:**
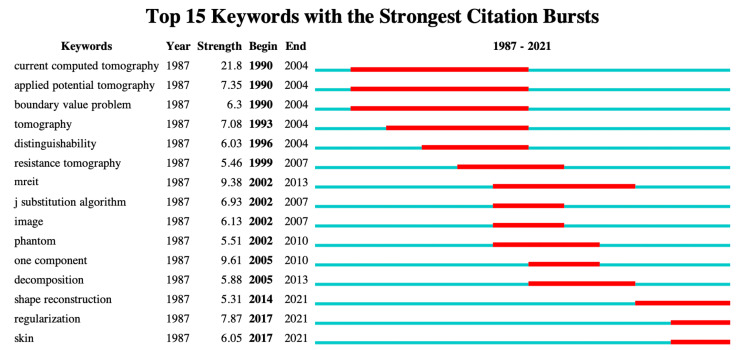
Top 15 highly burst keywords of EIT algorithm research between January 1, 1987, and December 31, 2021 The blue bars represented years in which the keyword gained a tender rise in co-occurrence, while the red bars represented sharp rises. EIT: Electrical impedance tomography. Image credits: Authors of this study.

The burst strength of keywords was another important indicator that reflected the research hotspots and emerging trends over time. Figure 5 presents the top 15 keywords with high burst values. Throughout the entire research period, "current computed tomography" (21.8) had the highest burst value, followed by "one component" (9.61), "mreit" (9.38), "regularization" (7.87), and "applied potential tomography" (7.35). In the earlier period of 1990-2013, keywords such as "current computed tomography," "one component," "mreit," "applied potential tomography," and "tomography" (7.08) were of significant concern. In contrast, "shape reconstruction" (5.31), "regularization" (7.87), and "skin" (6.05) gained strong attention after 2013 and maintained a high burst value until the present.

**Table 6:** Top 10 frequent keywords in the earliest and latest stages of EIT algorithm publications *Avg. pub. year: The average publication year of the articles in which the keyword occurs (to the nearest two decimal places). EIT: Electrical impedance tomography; MREIT: Magnetic resonance electrical impedance tomography.

	Earliest			Latest		
Rank	Keyword	Occurrences	Avg. pub. year*	Keyword	Occurrences	Avg. pub. year*
1	Applied potential tomography	25	2003.08	Voltage measurement	16	2020.50
2	Impedance imaging	19	2004.53	Deep learning	17	2020.06
3	Current computed tomography	94	2005.38	Convolutional neural network	6	2019.67
4	Uniqueness theorem	15	2005.60	Inclusion boundary reconstruction	5	2019.60
5	Kalman filter	24	2006.92	Acousto-electric tomography	5	2019.20
6	Boundary-value problem	48	2007.64	Skin	14	2019.14
7	J-substitution algorithm	36	2007.83	Strain	14	2019.08
8	Prior information	25	2007.88	Framework	8	2018.88
9	MREIT	41	2008.32	Spatial prior	6	2018.83
10	Conductivity image	22	2008.50	Machine learning	21	2018.81

Clustering and Time Evolution of Co-cited References

**Figure 6:**
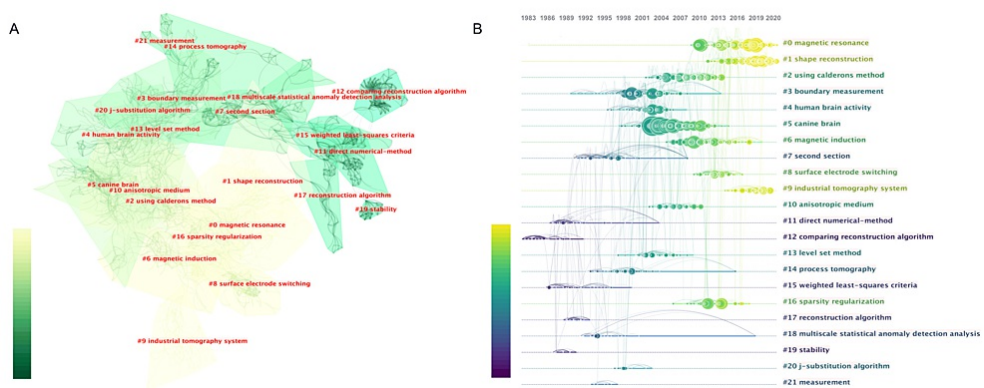
The clustering (A) and time-evolving analysis (B) of co-cited reference on EIT algorithm research between January 1, 1987, and December 31, 2021 Different colors meant the decadal transition of publication year. The vertical descending sequence represented the size of the cluster. (A) The line boldness represented the linkage tightness. (B) The color curves represented co-cited links added in the corresponding color year. Large nodes represented that the articles are either highly referenced, have reference bursts, or both. The specific publication year is indicated above the map. The modularity Q score is 0.8567, the mean silhouette is 0.9363, and the harmonic mean (Q, S) is 0.8947. EIT: Electrical impedance tomography. Image credits: Authors of this study.

References co-cited by publications on the EIT algorithm were divided into 22 clusters (described as cluster no, cluster size) (Figure 6), A vertical descending sequence illustrates the size of each cluster. The largest cluster, labeled as "magnetic resonance" (#0, 149), is followed by the next five largest clusters: "shape reconstruction" (#1, 137), "using Calderon's method" (#2, 121), "boundary measurement" (#3, 112), "human brain activity" (#4, 85), and "canine brain" (#5, 84). The largest eight clusters are listed in Appendix Table 6. Analysis of co-cited references over time revealed that references related to "boundary measurement" (#3), "human brain activity" (#4), "canine brain" (#5), and "second section" (#7) were highly cited before 2013, while "magnetic resonance" (#0) and "shape reconstruction" (#1) remained popular citation topics up to the present.

Co-cited References Burst Analysis

**Figure 7:**
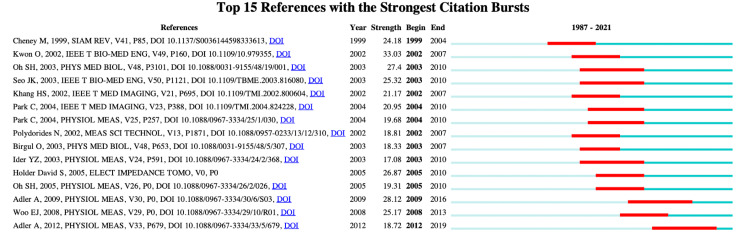
The top 15 references with the highest burst value of EIT algorithm research between January 1, 1987, and December 31, 2021 The dark blue bars showed years in which keywords gained a tender rise in citations, while the red bars showed sharp rises. EIT: Electrical impedance tomography. Image credits: Authors of this study.

The top 15 references with strong co-cited burst values of the EIT algorithm are summarized in Figure 7. These references started to burst in 1999, and approximately one-third of them were published in the journal *Physiol Meas*. The article "Cheney et al., 1999, SIAM Rev [13]" had the highest burst strength with a value of 33.03, followed by the article "Adler et al., 2009, *Physiol Meas* [17]" with a value of 28.12, the book "Holder, 2005, *Biomed Eng Online* [22]" with a value of 26.87, and the article "Seo, 2003, *IEEE Trans Biomed Eng* [23]" with a value of 25.32. The burst value duration ranged from four to seven years, and the most recent reference with a burst duration from 2012 to 2019 is the review by Adler et al. [24]. A summary of the top 15 co-cited references can be found in Appendix Table 7.

Discussion

The EIT image reconstruction algorithm plays a crucial role in its clinical application. Our bibliometric analysis has visualized the development process, current status, and emerging trends in EIT algorithm research. This analysis offers concise and informative references for researchers interested in the EIT algorithm, providing research directions and potential collaboration opportunities.

The number of annual articles on the EIT algorithm has steadily increased since its first publication by Rose and Cheney [25] in 1987, rising from one article to 140 articles in 2021. The USA, UK, China, and South Korea have contributed to over 60% of these articles. The significant rise in Chinese publications since 2015 may be attributed to the increasing interest from Chinese research institutes, such as Nanjing University of Aeronautics and Astronautics and Tsinghua University, inspired by the annual conference held by the EIT branch of the Chinese Society of Biomedical Engineering [26].

Notably, strong collaboration was observed among highly prolific institutions and authors (Figure 3). In contrast to the geographical distribution of cooperation projects in other academic fields, as reported by Kudu and Danış[27], researchers in the field of EIT actively collaborated across continents in the development of algorithms. For instance, the Graz Consensus Reconstruction Algorithm for EIT (GREIT), a consensus linear reconstruction algorithm for lung EIT, was developed through cooperation between Europe and America [17]. Similarly, researchers in Asia and America proposed an isotropic conductivity reconstruction algorithm in magnetic resonance electrical impedance tomography (MREIT), which is based on a single current injection to decrease scanning time [28]. *Physiol Meas* is the journal that has published the highest number of articles and received the most citations, possibly because of its long-standing association with the EIT Annual Conference and its publication of annual focus sets aimed at presenting the latest advances in EIT research. This association offers an opportunity for transnational and interest-driven cooperation on EIT algorithm research.

Several studies that pioneered classic EIT algorithm frameworks in the early stages were highly cited, which aligns with our frequently used keywords, such as "inverse problem" and "regularization." Vauhkonen et al. developed the utilization of Tikhonov regularization and prior information to enhance the stability and accuracy of EIT reconstruction [19]. Grychtol et al. proposed the use of a linear EIT reconstruction technique called GREIT for pulmonary monitoring. They achieved several important improvements, including a uniform amplitude response, minimized position error, reduced ringing artifacts, maintained uniform resolution, limited shape deformation, and provided high-resolution results [29]. These empirical EIT algorithms laid a solid foundation for medical applications. Subsequently, the primary objective of developing EIT algorithms has been to obtain the ideal solution to the inverse problem, ensuring that the measured boundary voltage maps the impedance distribution accurately. Initial studies focused on applying well-known theories for solving the inverse problem, such as the "uniqueness theorem," "Kalman filter," and "boundary-value problem." More recently, there has been an emerging trend in building EIT algorithms using artificial intelligence methods, such as "deep learning" [30], "convolutional neural networks" [31], and "machine learning" (Table 6) [32].

Bursting keywords over time illustrate the changes in the study topics. Initially, the points focused on fundamental issues of the EIT algorithm, specifically the resolution of the "boundary-value problem" and the enhancement of EIT distinguishability. Woo's research team presented a promising approach to achieve high-resolution impedance imaging by combining MRI and EIT. They incorporated the internal current density distribution into EIT image reconstruction, known as the "J-substitution algorithm" [33]. During the same period, there was a focus on developing accurate reconstruction models that incorporate anatomical structure and impedance distribution based on CT/MRI to improve spatial resolution, shape error, and position error [34]. Additionally, there were studies on accurate phantoms being conducted concurrently to evaluate the efficacy of the new EIT algorithm. Currently, the research focus has shifted to improving custom reconstruction strategies that meet specific application requirements. For instance, Liu et al. approached the EIT reconstruction problem as a "shape reconstruction" problem, aiming to enhance the tolerance toward modeling errors and uncertainties in EIT for lung imaging [35].

The co-cited reference cluster highlights several methods related to the EIT algorithm, including weighted least-squares criteria, Calderon's method, the level set method, sparsity regularization, and the J-substitution algorithm. It also mentions that "shape reconstruction" has been the focal point of research since 2013. Novel reconstruction strategies are often proposed to meet both the advanced acknowledgment of inverse problem theories and clinical requirements. The review by Cheney et al. from the group of Rensselaer Polytechnic Institute, published in 1999, was the highest burst co-cited reference [13]. This review describes the design of the famous Rensselaer EIT system, known as the adaptive current tomography (ACT) system, and surveys typical reconstruction algorithms. The time evolution of co-cited references further shows that "MREIT" novel imaging modalities regarding magnetic resonance is a highly co-cited reference cluster. Since Woo's research group proposed MREIT in 2005 [36], articles regarding innovative MREIT algorithms and their validation were published, providing the basis for future generations to establish the basic algorithm framework of MREIT. In summary, high-quality reviews and articles from pioneering institutions over time provide a relatively complete knowledge map at that time. Integrating EIT with other imaging technologies may open up a new research branch for EIT algorithms.

Strengths and limitations

This study aims to visualize the current situation, hot issues, and research trends in EIT algorithm research between 1987 and 2021, providing a convenient and objective reference for researchers in need. However, there are certain limitations. First, due to the interdisciplinary nature of EIT, we chose to include all citation indexes under WoSCC and subjectively excluded less relevant literature with the help of EIT experts to ensure the maximum inclusion of relevant literature. Second, the WoSCC database is constantly updated, so some new data may be missed, even if the entire database search was conducted within a single day. Third, the various ways in which authors, institutions, and keywords are expressed result in a dispersion of counts and clusters. Although these issues were addressed using the merge and normalization function of the software, they cannot be eliminated.

## Conclusions

The EIT algorithm has been the subject of research over the past three decades. The research focus on this topic has generally progressed through three stages: first, the establishment of basic algorithm frameworks; second, the optimization of algorithms to enhance imaging performance; and lastly, the improvement to facilitate clinical applications. Another emerging research trend is the exploration of more diversified algorithms and the intersection of EIT with machine learning techniques.

## Appendices

**Table 7:** Major keywords in each keyword co-occurrence cluster on the EIT algorithm EIT: Electrical impedance tomography; MREIT: Magnetic resonance electrical impedance tomography.

Co-occurrence cluster	Keywords
Cluster 1: Algorithm	fidelity-embedded regularization; regularization; inverse problem; electrode models; finite element method; optimization; state estimation; Kalman filter; machine learning; level set method; approximation errors; deep learning; statistical inversion; Bayesian inversion; extended Kalman filter; sensitivity-analysis; total variation regularization; boundary element method; conformal transformation; modeling errors; Tikhonov; adaptive mesh refinement; boundary estimation; Calderon's method; iteration method; Monte-Carlo methods; nonlinear image-reconstruction; singular value decomposition; sparse representation.
Cluster 2: Lung and brain-based clinical application direction	ventilation; stroke; volume; perfusion; regional lung ventilation; recruitment; brain-function; end-expiratory pressure; hemorrhage; human brain-function; lung perfusion; stroke volume; current patterns; derecruitment; infants; epilepsy; GREIT; multifrequency EIT system; source localization; acute respiratory distress syndrome; blood-flow.
Cluster 3: New algorithm reconstruction methods	MREIT; b-z algorithm; J-substitution algorithm; magnetic flux density; contrast; ultrasound; magnetic induction; anisotropy; thermoacoustic tomography; current-density distribution; ablation; conductivity tensor; d-bar method.
Cluster 4: Key problems in the reconstruction algorithm	artefact; inverse conductivity problem; uniqueness; boundary-value problem; convergence; Calderon problem; Tikhonov regularization; stability; prior information; ill-posed problems; Neumann-to-Dirichlet map; level set; nonlinear inverse problems; shape optimization; minimization; convergence-rates; Lipschitz stability; topology optimization; imperfectly known boundary.
Cluster 5: Optimization in the algorithm	factorization method; identification; boundary measurements; shape reconstruction; asymptotic formulas; hybrid imaging; small-diameter; linear sampling method; optical tomography; elliptic problems; inverse scattering; monotonicity method; location; acousto-electric tomography; current projection method; microwave imaging.
Cluster 6: Extrapulmonary clinical application	breast cancer; cancer detection; diagnosis; mammography; prostate cancer; intracerebral hemorrhage.
Cluster 7: Electromagnetic induction	forward problem; hardware; electromagnetic tomography; magnetic induction tomography (MIT); phantoms; sensitivity maps.
Cluster 8: Others	inclusion boundary reconstruction.

**Table 8:** The largest eight clusters in co-cited reference on the EIT algorithm LSI: Latent semantic indexing; LLR: Log-likelihood ratio; MI: Mutual information; EIT: Electrical impedance tomography.

Cluster ID	Size	Silhouette	Label (LSI)	Label (LLR)	Label (MI)	Average Year
0	149	0.883	Dynamic brain	Magnetic resonance (505.63, 1.0E-4)	Measurement (3.2)	2014
1	137	0.942	Nonlinear reconstruction	Shape reconstruction (579.83, 1.0E-4)	Measurement (0.88)	2017
2	121	0.904	D-bar method	Using Calderon’s method (936.56, 1.0E-4)	Quasistatic reconstruction (1.43)	2007
3	112	0.905	Factorization method	Boundary measurement (520.3, 1.0E-4)	Reconstructing isotropic conductivities (0.24)	2000
4	85	0.946	Human brain activity	Human brain activity (440.04, 1.0E-4)	Tomography (0.32)	2001
5	84	0.952	Magnetic resonance	Magnetic resonance (1913.35, 1.0E-4)	Inductive magnetic resonance (0.66)	2004
6	81	0.968	Magnetic induction	Magnetic induction (808.48, 1.0E-4)	Measurement (0.34)	2010
7	65	0.917	Nonlinear reconstruction	Second section (146.24, 1.0E-4)	Finite element method (0.02)	1994

**Table 9:** Top 15 references with the strongest citation bursts

Rank	Title	Type	Author	Year	Journal	Strength	Reference
1	Electrical impedance tomography	Article	Cheney et al.	1999	Siam Rev	24.18	[13]
2	Magnetic resonance electrical impedance tomography (MREIT): simulation study of J-substitution algorithm	Article	Kwon et al.	2002	IEEE T Bio-Med Eng	33.03	[37]
3	Conductivity and current density image reconstruction using harmonic Bz algorithm in magnetic resonance electrical impedance tomography	Article	Oh et al.	2003	Phys Med Biol	27.4	[38]
4	Reconstruction of conductivity and current density images using only one component of magnetic field measurements	Article	Seo et al.	2003	IEEE T Bio-Med Eng	25.32	[23]
5	J-substitution algorithm in magnetic resonance electrical impedance tomography (MREIT): phantom experiments for static resistivity images	Article	Khang et al.	2002	IEEE T Med Imaging	21.17	[33]
6	Electrical conductivity imaging using gradient B/sub z/ decomposition algorithm in magnetic resonance electrical impedance tomography (MREIT)	Article	Park et al.	2004	IEEE T Med Imaging	20.95	[39]
7	Static conductivity imaging using variational gradient Bz algorithm in magnetic resonance electrical impedance tomography	Article	Park et al.	2004	Physiol Meas	19.68	[40]
8	A Matlab toolkit for three-dimensional electrical impedance tomography: a contribution to the Electrical Impedance and Diffuse Optical Reconstruction Software project	Article	Polydorides et al.	2002	Meas Sci Technol	18.81	[41]
9	Current constrained voltage scaled reconstruction (CCVSR) algorithm for MR-EIT and its performance with different probing current patterns	Article	Birgul et al.	2003	Phys Med Biol	18.33	[42]
10	Uniqueness and reconstruction in magnetic resonance–electrical impedance tomography (MR-–EIT)	Article	Ider et al.	2003	Physiol Meas	17.08	[43]
11	Electrical impedance tomography (1st edition)	Book	Wang G	2005	Institute of Physics Publishing, Bristol and Philadelphia	26.87	[22]
12	Electrical conductivity images of biological tissue phantoms in MREIT	Article	Oh et al.	2005	Physiol Meas	19.31	[36]
13	GREIT: a unified approach to 2D linear EIT reconstruction of lung images	Article	Adler et al.	2009	Physiol Meas	28.12	[17]
14	Magnetic resonance electrical impedance tomography (MREIT) for high-resolution conductivity imaging	Review	Woo and Seo	2008	Physiol Meas	25.17	[44]
15	Whither lung EIT: Where are we, where do we want to go and what do we need to get there?	Review	Adler et al.	2012	Physiol Meas	18.72	[24]
